# Retraction: Silencing of SOX12 by shRNA suppresses migration, invasion and proliferation of breast cancer cells

**DOI:** 10.1042/BSR-2016-0053_RET

**Published:** 2023-01-19

**Keywords:** breast cancer, cell cycle arrest, invasion, migration, proliferation, SOX12

This Retraction follows an Expression of Concern relating to this article previously published by Portland Press. This article is being retracted from *Bioscience Reports* at the request of the authors, following receipt of a notification from a reader alerting the Editorial Office to similarities that exist between the Figure 5C Cyclin/BT474 western blot bands from this article and the Figure 5C B-Carwnin/HO-8910 bands from a paper by Cheng et al. 2015 (doi: 10.18632/oncotarget.4541). During our investigation, the GAPDH bands from figure 3D were also highlighted by a reader as being very similar to those found in Figure 1E from Guo et al. 2017 (https://doi.org/10.1038/s41598-017-01832-y) and Figure 6E of Zheng et al. 2020 (https://doi.org/10.1186/s13046-019-1504-5). The authors were unable to provide the requested original data and responded to our queries by repeating the experiment, providing the Editorial Office with the new data. However, the Editorial Board did not feel that an adequate explanation to the duplicated blots was provided and remain concerned about the lack of original data. The authors therefore wish to retract the article, and the Editor-in-Chief and Editorial Board agree with the retraction.

